# The Immune Checkpoint Protein PD-L1 Regulates Ciliogenesis and Hedgehog Signaling

**DOI:** 10.3390/cells13121003

**Published:** 2024-06-08

**Authors:** Ewud Agborbesong, Xiaogang Li

**Affiliations:** 1 Department of Internal Medicine, Mayo Clinic, Rochester, MN 55905, USA; 2 Department of Biochemistry and Molecular Biology, Mayo Clinic, R, 200 1st Street, SW, Rochester, MN 55905, USA

**Keywords:** cell biology, PD-L1, primary cilia, cilia protein trafficking

## Abstract

The primary cilium, an antenna-like sensory organelle that protrudes from the surface of most eukaryotic cell types, has become a signaling hub of growing interest given that defects in its structure and/or function are associated with human diseases and syndromes, known as ciliopathies. With the continuously expanding role of primary cilia in health and diseases, identifying new players in ciliogenesis will lead to a better understanding of the function of this organelle. It has been shown that the primary cilium shares similarities with the immune synapse, a highly organized structure at the interface between an antigen-presenting or target cell and a lymphocyte. Studies have demonstrated a role for known cilia regulators in immune synapse formation. However, whether immune synapse regulators modulate ciliogenesis remains elusive. Here, we find that programmed death ligand 1 (PD-L1), an immune checkpoint protein and regulator of immune synapse formation, plays a role in the regulation of ciliogenesis. We found that PD-L1 is enriched at the centrosome/basal body and Golgi apparatus of ciliated cells and depleting PD-L1 enhanced ciliogenesis and increased the accumulation of ciliary membrane trafficking proteins Rab8a, BBS5, and sensory receptor protein PC-2. Moreover, PD-L1 formed a complex with BBS5 and PC-2. In addition, we found that depletion of PD-L1 resulted in the ciliary accumulation of Gli3 and the downregulation of Gli1. Our results suggest that PD-L1 is a new player in ciliogenesis, contributing to PC-2-mediated sensory signaling and the Hh signaling cascade.

## 1. Introduction

The primary cilium is a microtubule-based structure that extends from the apical surface of most eukaryotic cells and functions as a sensory organelle, sensing and transducing environmental cues through ciliary sensory receptors, such as the polycystins [1,2]. Primary cilia are critical for normal development and homeostasis [3,4,5]. As such, defects in the formation and/or function of primary cilia are associated with a wide range of human inherited multisystemic disorders, including polycystic kidney disease (PKD) and Bardet–Biedl syndrome (BBS), referred to as ciliopathies [6,7]. Despite the physiological and clinical relevance of primary cilia, the molecular mechanisms that regulate cilia biogenesis and function are not completely understood.

The formation and maintenance of the axoneme, the most prominent feature of the cilia, is dependent on the recycling of the bidirectional intraflagellar transport (IFT) machinery along the axonemal microtubules. This machinery consists of the anterograde transport (IFT-B) and the retrograde transport (IFT-A) complexes proposed to carry ciliary proteins to, and from the cilia tip [8]. An IFT cargo adaptor, the BBSome complex, coordinates IFT-A/B complex trafficking associated with membrane protein removal from the cilia via retrograde IFT [9]. The BBSome trains, for example, were reported to remove activated G-protein-coupled receptors from the cilia via the transition zone [10]. Trafficking of ciliary proteins, including membrane receptors, must be precisely regulated and the proteins properly located for cilia to exhibit their specific sensory properties and execute their cellular functions [11,12]. As such, the primary cilium has a sorting mechanism to selectively regulate the entry and exit of various ciliogenic proteins. In the Hedgehog (Hh) signaling pathway, for example, the dynamic movement of key components, such as Smoothened (Smo), and Gli-Kruppel family of zinc-finger (Gli) transcription factors into and out of the cilia rely significantly on the IFT machinery [13,14,15]. Upon activation of the Hh pathway, Smo accumulates in the cilium, resulting in the cilia accumulation of Gli2/3 associated with the production of Gli activators [16]. Membrane trafficking regulators such as the BBS proteins and the ras-related GTP-binding protein 8 (Rab8) also collaborate with the IFT machinery to control ciliary membrane biogenesis and the trafficking of membrane proteins to the cilium [11,17,18]. The Golgi apparatus, which spreads all over the cell and is particularly enriched at the primary cilium base, plays an essential role in intracellular sorting, trafficking, and the targeting of proteins to specific cellular compartments [19,20]. Alterations in Golgi-associated proteins have also been suggested to modulate ciliogenesis [21].

The similarities between the primary cilium and immune synapse (interface formed between an antigen-presenting cell and the effector immune cell) have been increasingly recognized [22]. First, centrosome polarization at the immune synapse in cytotoxic T lymphocytes (CTL) resembles the centriole docking process during the initiation of cilia assembly [22,23]. Second, several centriole proteins such as CEP83, CEP97 and CP110 [24], localize at the immune synapse in CTL [25]. Third, both primary cilia and immune synapse formation involve actin reorganization. In immune synapse assembly, the BBSome complex protein BBS1 controls T cell polarity by allowing for centrosome polarization towards the immune synapse and clearance of centrosomal F-actin [26]. The BBSome and IFT-B complexes collaborate during primary cilia assembly to modulate the trafficking of ciliary cargoes. Additionally, there are similarities in the trafficking mechanism of immunological synapses and primary cilia. IFT proteins, such as Ift20 typically associated with primary cilia [27], are reported to modulate T lymphocyte activation [28]. Lastly, both the primary cilium and the immune synapse exhibit concentrated signal transduction activities. At the immune synapse, signaling molecules accumulate in specific regions like the supramolecular activating complex at the center of the synapse, to facilitate efficient T-cell signaling [29,30]. In the primary cilium, the ciliary membrane is enriched with receptors that receive and transmit a variety of signals from the extracellular environment [31], permitting the primary cilium to function as a central signaling hub within the cell. Considering the morphological and functional homology between primary cilia and the immune synapse, it is interesting to point out that cilia-associated proteins have been found to play various roles in immune synapse formation [32]. However, whether immune-synapse-associated proteins play a role in primary cilium formation and/or function to the best of our knowledge is unknown.

Programmed death-1 ligand (PD-L1; also referred to as B7-H1 and CD279), is a transmembrane glycoprotein that is usually expressed by immune cells including activated T cells and B cells, macrophages, dendritic cells, and by some epithelial cells [33]. PD-L1 is also expressed by tumor cells, where it acts as an adaptive immune mechanism, enabling immune tolerance and preventing auto-immune responses by delivering intrinsic intracellular signals that regulate stress response and enhance cancer cell survival [34]. PD-L1 has been reported to influence immune response by decreasing T-cell motility and promoting stable immune synapse formation [35,36]. Aside from its immune response functions, PD-L1 has also been shown to exert other roles in a variety of tumor cell types. For example, PD-L1 has been reported to promote glycolytic metabolism in a variety of tumor cells [37] and induce epithelial-to-mesenchymal transition (EMT) in renal cancer cells [38]. Considering the role of PD-L1 in regulating immune synapse formation, and the striking similarities between immune synapse and the primary cilia, it is possible that PD-L1 might have a role in regulating primary cilia formation. 

In this study, we investigated the role of PD-L1 in the regulation of ciliogenesis. We show that PD-L1 is located at the Golgi complex and centrosome in ciliated cells and that its depletion enhanced cilia formation and function by (i) modulating the ciliary accumulation of proteins associated with cilia assembly and maintenance; (ii) modulating the ciliary accumulation of the cilia sensory receptors; and (iii) interacting with ciliary membrane trafficking and sensory receptor proteins. In addition, we found that PD-L1 plays a potential role in the regulation and/or maintenance of the centrosome integrity and mitotic/cytokinetic cell division by modulating centrosome duplication and chromosome segregation. Our data reveal how PD-L1 is co-opted as a regulator of cilia signaling and establishes a perspective relationship between PD-L1 and genome instability.

## 2. Materials and Methods

### 2.1. Cell Culture

NIH3T3 (ATCC, Cat# CRL-1658) fibroblast, mIMCD3 (ATCC, Cat# CRL-2123) and HEK293T (ATCC, Cat# CRL-3216) cells were maintained at 37 °C in 5% CO_2_ in DMEM (Invitrogen, Waltham, MA, USA) supplemented with 10% FBS (Sigma, St. Louis, MO, USA) and 1% Pen Strep (Gibco, Grand Island, NY, USA). RCTE (ATCC, Cat# PCS-400-010) and RPE (ATCC, Cat# CRL-4000) cells were maintained at 37 °C in 5% CO_2_ in DMEM/F-12, GlutaMAX™ (Gibco, Grand Island, NY, USA) supplemented with 10% FBS and 1% Pen Strep. For analysis of ciliary assembly, cells were plated at 50–60% confluence in plates containing glass coverslips, grown to approximately 80% to complete confluency, and starved for 24–48 h (regular DMEM or DMEM/F-12 without serum) to induce cilia growth, followed by immunostaining. For SAG experiments, NIH3T3 cells were plated at approximately 80% confluent densities and serum starved for 48 h. SAG (Calbiochem, San Diego, CA, USA) was used at 500 nM.

### 2.2. Plasmids

The GFP-tagged PD-L1 and Myc-tagged PC-2 plasmids were purchased from Addgene (Watertown, MA, USA).

### 2.3. Antibodies and Reagents

Primary antibodies used in this study are listed as follows: mouse monoclonal antibodies against acetylated α-tubulin (6-11B-1, Sigma, St. Louis, MO, USA, T7451, 1:4000 used for immunofluorescence [IF]), γ-tubulin (GTU-88, Sigma, T5326, 1:1000 used for IF), α-tubulin (DM1A, sc-32293, 1:1000), Ninein (F-7, Santa Cruz, Dallas, TX, USA, sc-390540, 1:300 for IF), Cep164 (E-9, Santa Cruz, sc-515403, 1:300 used for IF), C-nap1 (E-9, Santa Cruz, sc-515403, 1:300 used for IF), Smo (E-5, Santa Cruz, sc-166685, 1:100 used for IF), Gli1 (C-1, Santa Cruz, sc-515751, 1:500 used for WB), Giantin (9B6, Abcam, Waltham, MA, USA, ab37266, 1:500 used for IF), actin (AC-15, Sigma, A1978, 1:3000 used for Western blot [WB]), GFP-tag (B-2, sc-9996, 1:1000 for WB), and Myc (9E10, sc-40, 1:1000 for WB); rabbit polyclonal antibodies against PD-L1 (Proteintech, Rosemont, IL, USA, 17952-1-AP, 1:300 used for IF, and 1:1000 used for WB), Cep135 (Abcam, ab75005, 1:300 used for IF), Ift140 (Proteintech, 17460-1-AP, 1:300 used for IF, and 1:1000 for WB), Ift20 (Proteintech, 13615-1-AP, 1:300 used for IF, and 1:1000 for WB), Rab8a (Proteintech, 55296-1-AP, 1:300 used for IF, and 1:1000 for WB), BBS5 (Proteintech, 14569-1-AP, 1:300 used for IF, and 1:1000 for WB), ARL13B (Proteintech, 17711-1-AP, 1:2000 for IF), GFP-tag (Proteintech, 50430-2-AP, 1:1000 for WB) and Myc-Tag (CST, 71D10, 1:1000 used for WB); goat polyclonal antibody against polycystin 2 (E-20, Santa Cruz, sc-10377, 1:500 for WB). Secondary antibodies used for IF staining include goat anti-mouse or donkey anti-rabbit conjugated with Alex Fluor 488 or 555, purchased from Invitrogen; and the secondary antibodies used for Western blot include donkey anti-rabbit IgG–horseradish peroxidase (sc-2313), donkey anti-goat IgG–horseradish peroxidase (sc-2020), and goat anti-mouse IgG–horseradish peroxidase (sc-2005), purchased from Santa Cruz Biotechnology Inc. (Dallas, TX, USA).

### 2.4. Cell Immunofluorescence Staining

Cells were grown on coverslips to the desired confluency and rinsed with 1× phosphate-buffered saline (PBS). The cells were then either fixed with 4% paraformaldehyde (PFA) (ThermoFisher Scientific, Rockford, IL, USA) for 10 min at 37 °C, followed by permeabilization with 0.2% Triton X-100 (Sigma, St. Louis, MO, USA) at room temperature for 10 min, or with cold 95% methanol for 10 min at −20 °C, followed by permeabilization with 0.2% Triton X-100 for 15 min at 37 °C. Cells were then washed 3× with PBS and blocked for 1 h in 2% BSA (Sigma, St. Louis, MO, USA). After blocking, cells were sequentially incubated with primary and secondary antibodies for 1 h.

### 2.5. Western Blot Analysis and Immunoprecipitation

Cells were homogenized in lysis buffer (20 mM Tris-HCl, pH 7.4, 150 mM NaCl, 10% glycerol, 1% Triton X-100, 1 mM Na3VO4, 25 mM β-glycerol-phosphate, 0.1 mM PMSF, Roche complete protease inhibitor set, and Sigma-Aldrich phosphatase inhibitor set, St. Louis, MO, USA), and centrifuged at 20,000× *g* for 20 min. Protein concentration was measured using the BCA Pierce Protein assay kit (ThermoFisher, Waltham, MA, USA) and normalized to the lowest concentration. Protein samples were subjected to standard SDS-PAGE gels, transferred to immuno-blot PVDF membranes (Millipore, Burlington, MA, USA), blocked with 10% nonfat dry milk, and then incubated overnight at 4 °C with primary antibodies. 

For immunoprecipitation, HEK293 cells expressing GFP-tagged PD-L1 or Myc-tagged PC-2 were lysed in a lysis buffer. The lysate was centrifuged for 20 min at 20,000× *g* at 4 °C, and then the supernatant was incubated with protein A agarose beads (Pierce, Appleton, WI, USA) and either anti-BBS5 (Proteintech, 14569-1-AP), anti-GFP-tagged GFP-tag (B-2, sc-9996) or anti-Myc-tagged (CST, 71D10) antibodies and their isotype control antibodies, in PBS containing 5 mg/mL BSA overnight at 4 °C on a rotating platform. The beads were washed 3×, and the immune complexes were eluted off the beads using a loading buffer and then separated by SDS–PAGE gels.

### 2.6. RNA Interference

The RNA oligonucleotides that specifically targeted mouse PD-L1 (sc-39700) and mouse BBS5 (sc-72165) were purchased from Santa Cruz Biotechnology Inc. The RNA oligonucleotides that specifically targeted human PD-L1 (Cat# L-015836-01-0005) were purchased from Dharmacon (Lafayette, CO, USA). The RNA oligonucleotides were transfected using Lipofectamine RNAiMAX (Invitrogen) following the manufacturer’s instructions. 48 h after transfection, cells were harvested and analyzed by Western blotting.

### 2.7. Real-Time Quantitative Reverse Transcription PCR (qRT-PCR)

Total RNA was extracted using the RNeasy Plus Mini Kit (QIAGEN, Germantown, MD, USA). Total RNA (1 μg) was reverse-transcribed using an iScript cDNA Synthesis Kit (Bio-Rad, Hercules, CA, USA) and amplified in triplicate using iTaq SYBR Green Supermix with ROX (Bio-Rad) with a real-time PCR machine (Bio-Rad), according to the manufacturer’s instructions. The expression levels of target genes were normalized to the expression level of actin or GAPDH.

### 2.8. Microscopy and Imaging

Images were acquired using an imaging microscope (Nikon TE 2000-U) with a Plan Apochromat 60× 1.49 oil objective (Nikon, Melville, NY, USA).

### 2.9. Statistics

All data are presented as mean ± SD. All statistical analyses were performed using SPSS Statistics 22 software and GraphPad Prism 9.1.0. Ciliary length and intensity measurements were performed using ImageJ (https://imagej.net/ij/, accessed on 5 June 2024). Statistical significance was determined by unpaired Student’s *t* test and 1-way ANOVA, and a *p* value of less than 0.05 was considered significant.

## 3. Results

### 3.1. PD-L1 Is Localized to the Centrosome/Basal Body and the Golgi Complex

**Figure 1:**
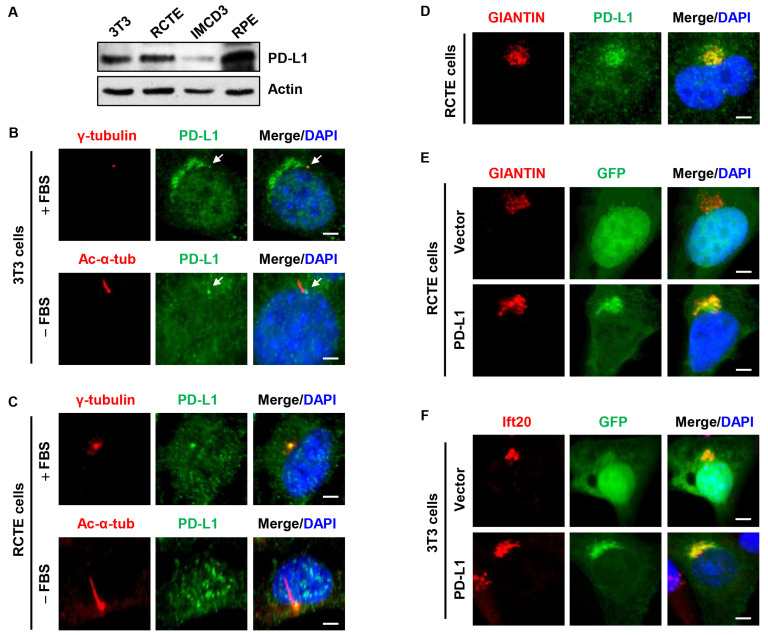
PD-L1 is located at the centrosome and Golgi. (**A**) Western blot analysis indicating the differential expression of PD-L1 in fibroblast and epithelial cells. (**B**) NIH3T3 cells stained with PD-L1 (green) antibody and co-stained with centrosome marker, γ-tubulin (red) (top panel), and cilium marker, acetylated-α-tubulin (red) (bottom panel). Centrosome localized PD-L1 is indicated with the white arrow. (**C**) RCTE cells stained with PD-L1 (green) antibody and co-stained with γ-tubulin (red) (top panel) and acetylated-α-tubulin (red) (bottom panel). Cells were serum starved to induce cilia growth. (**D**) RCTE cells stained with PD-L1 (green) antibody and co-stained with Golgi marker, GIANTIN (red). (**E**) Overexpression of GFP-PD-L1 (green) co-stained with Golgi marker, Giantin (red) in RCTE cells. (**F**) Overexpression of GFP-PD-L1 (green) co-stained with Golgi-associated protein Ift20 (red) in NIH3T3 cells. All cells were counterstained with DAPI (blue). Scale bars, 20 μm.

To investigate the potential role of PD-L1 in the regulation of ciliogenesis, first, we evaluated cells known to grow primary cilia and found that PD-L1 was differentially expressed in NIH3T3 fibroblast cells, human renal cortical epithelial cells (RCTE), mouse inner medullary collecting duct 3 cells (IMCD3), and human retinal pigment epithelial cells (RPE1) (Figure 1A). The immunofluorescence staining showed that a sub-population of endogenous PD-L1 was localized at the centrosome/basal body and cilia base characterized by its co-localization with γ-tubulin, a centrosome marker, and its location at the base of cilia marked by acetylated α-tubulin (Ac-α-tub) staining in NIH3T3 cells (Figure 1B), RCTE cells (Figure 1C), and mouse IMCD3 cells (Supplemental Figure S1A). In addition, PD-L1 also overlapped with known centrosome markers, including Cep164 (centrosomal protein 164), ninein, and C-nap1 (also known as Cep250) (Supplemental Figure S1B). Furthermore, we found that endogenous PD-L1 had a stacked peri-nuclear, Golgi-like structure in NIH3T3 cells (Figure 1B), RCTE cells (Figure 1D) and RPE cells (Supplemental Figure S1C) co-stained with the known Golgi marker GIANTIN. Exogenously expressed GFP-tagged PD-L1 (GFP-PD-L1) also accumulated at the Golgi region when co-stained with γ-tubulin in NIH3T3 and IMCD3 cells (Supplemental Figure S1D) and overlapped with GIANTIN in RCTE cells (Figure 1E) and RPE cells (Supplemental Figure S1E). In addition, GFP-PD-L1 partially overlapped the known Golgi-associated intraflagellar transport protein 20 (Ift20) in NIH3T3 cells (Figure 1F). Collectively, these results suggest that PD-L1 may be a centrosome protein yet an intrinsic component of the Golgi complex.

To confirm that PD-L1 is an intrinsic component of the Golgi complex, we examined the fate of PD-L1 upon treatment with nocodazole. Nocodazole causes depolymerization of microtubules in cells which eventually leads to the dispersal of Golgi elements throughout the cytoplasm [39,40]. Upon treatment with nocodazole, we found that PD-L1 dispersed from its compact peri-nuclear structure to small puncta distributed throughout the cytoplasm (Supplemental Figure S2A). However, the compact peri-nuclear structure began to reform when the nocodazole was washed out and the cells were re-fed with a normal culture medium (Supplemental Figure S2A). Next, we evaluated whether PD-L1 may affect the Golgi structure. We found that knockdown of PD-L1 with siRNA (Supplemental Figure S2B) exhibited no change in the localization and intensity of the Golgi marked by GIANTIN in RCTE cells (Supplemental Figure S2C,D). These results suggest that PD-L1 is associated with the Golgi complex and its depletion has no effect on Golgi structure.

### 3.2. PD-L1 Segregates with the Spindle Poles and Affects Mitosis and Cytokinesis

To examine the fate of PD-L1 during the cell cycle, actively growing cells were stained for PD-L1. We observed that PD-L1 segregated with the spindle poles during mitosis as indicated by the co-staining of α-tubulin and PD-L1 (Supplemental Figure S3A), and γ-tubulin and PD-L1 (Supplemental Figure S3B), suggesting that PD-L1 may play a role in cell division. To gain insights into this potential role of PD-L1 in cell division, we evaluated whether knockdown of PD-L1 in NIH3T3 cells (Supplemental Figure S3C) affects cell cycle profile by flow cytometry analysis. We found that knockdown of PD-L1 did not significantly affect the percentage of cells in the G1, S, and G2/M phases of the cell cycle compared to control cells (Supplemental Figure S3D). Interestingly, we found that knockdown of PD-L1 resulted in a significant increase in abnormal centrosome amplification (≥3 centrosomes per cell), and the increased formation of micronuclei (Supplemental Figure S3E,F) compared to control NIH3T3 cells. These results suggest that PD-L1 contributes to the maintenance of centrosome integrity and chromosome instability. 

### 3.3. PD-L1 Regulates Ciliogenesis

**Figure 2:**
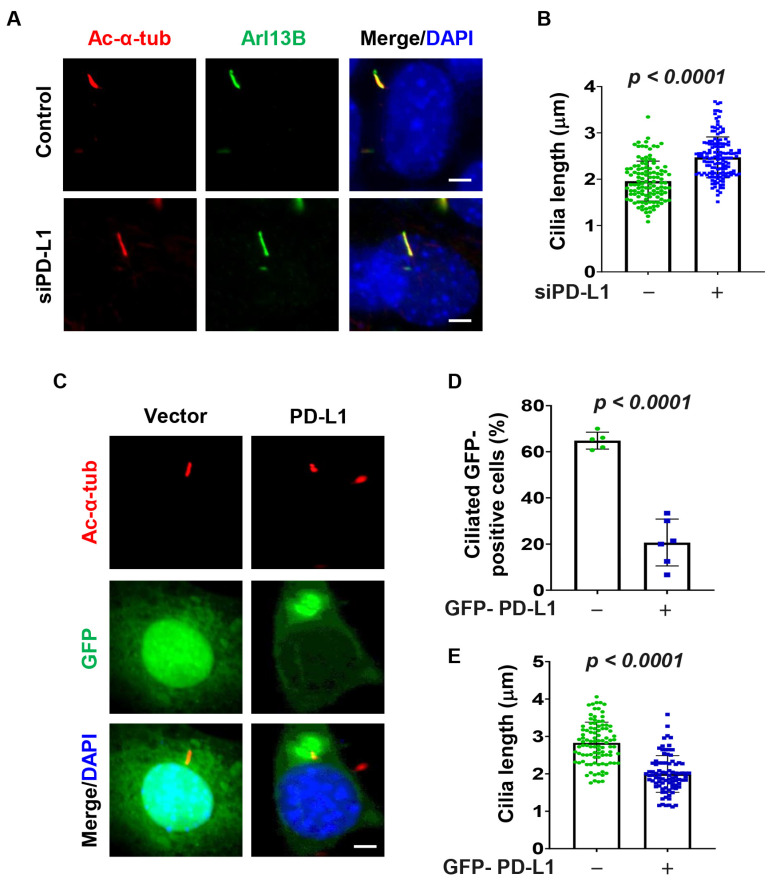
PD-L1 regulates ciliogenesis in 3T3 cells. (**A**) Representative images of acetylated-α-tubulin (red) co-stained with Arl13B (green) in PD-L1 siRNA knockdown NIH3T3 cells compared to control siRNA cells. (**B**) Quantitative data of cilium length (*n* > 100) in PD-L1 siRNA knockdown NIH3T3 cells compared to control siRNA cells. (**C**–**E**) Representative images of acetylated-α-tubulin (red) and GFP-PD-L1 (green) (**C**), and quantitative data of percentage ciliated cells (*n* > 75) (**D**), and cilium length (*n* > 75) (**E**), in NIH3T3 cells transfected with GFP-PD-L1. All cells were counterstained with DAPI (blue). The quantitative data was obtained by analyzing only cells that were GFP-positive. Scale bar, 20 μm.

To determine the role of PD-L1 on ciliogenesis, we knocked down PD-L1 with siRNA and found that depletion of PD-L1 (Supplemental Figure S4A) resulted in an increase in the average cilia length in serum-starved NIH3T3 cells as visualized by cilia markers Ac-α-tub and ADP-ribosylation factor-like 13b (Arl13b) (Figure 2A,B). We also found that overexpression of GFP-PD-L1 in NIH3T3 cells decreased the percentage of ciliated cells and the average cilia length compared to GFP-vector transfected control cells (Figure 2C–E). To further confirm the role of PD-L1 on ciliogenesis, we evaluated its effect on ciliogenesis in RCTE and RPE cells. We found that depletion of PD-L1 also resulted in an increase in the average cilia length in RCTE cells (Supplemental Figure S4B,C) and RPE cells (Supplemental Figure S4D–F). Together, these results suggest that PD-L1 contributes to cilia assembly and/or maintenance.

### 3.4. Depletion of PD-L1 Increases Golgi Accumulation of Ift20, and Ciliary Rab8a and BBS5

**Figure 3:**
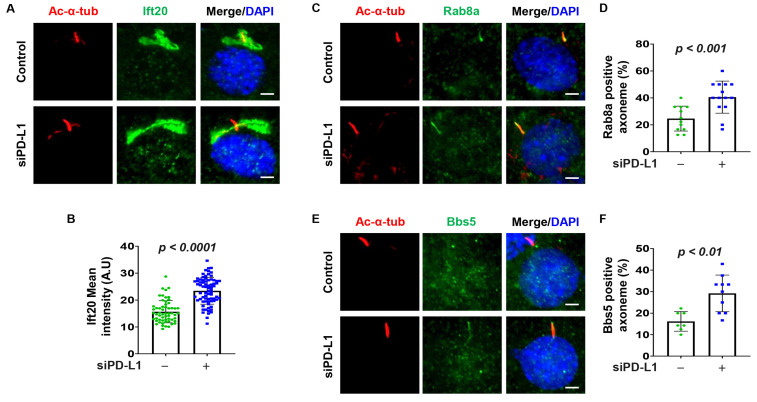
PD-L1 affects the Golgi accumulation of Ift20 and ciliary protein trafficking of Rab8a and BBS5. (**A**) NIH3T3 cells co-stained for acetylated-α-tubulin and Ift20 (green) in PD-L1 siRNA knockdown NIH3T3 cells compared to control siRNA cells. (**B**) Quantitative data of fluorescence intensity of Ift20 in PD-L1 siRNA knockdown NIH3T3 cells compared to control siRNA cells (*n* > 75). (**C**) NIH3T3 cells co-stained for acetylated-α-tubulin and the small GTPase Rab8a (green), in PD-L1 siRNA knockdown NIH3T3 cells compared to control siRNA cells. (**D**) Quantitative data of Rab8a-positive cilia in PD-L1 siRNA knockdown NIH3T3 cells compared to control siRNA cells (*n* > 75). (**E**) NIH3T3 cells co-stained for acetylated-α-tubulin and BBS5 (green), in PD-L1 siRNA knockdown NIH3T3 cells compared to control siRNA cells. (**F**) Quantitative data of BBS5-positive cilia in PD-L1 siRNA knockdown NIH3T3 cells compared to control siRNA cells (*n* > 60). All cells were counterstained with DAPI (blue). Scale bar, 20 μm.

Studies have indicated that Ift20, a subunit of the IFT complex crucial for ciliogenesis, and a Golgi-associated protein, plays a role in trafficking of ciliary membrane proteins from Golgi to the cilium [27]. The co-localization of GFP-PD-L1 and Ift20 prompted us to investigate whether PD-L1 regulates ciliogenesis by modulating the trafficking of cilia proteins. First, we found that depletion of PD-L1 with siRNA increased the intensity of Ift20 at the Golgi (Figure 3A,B). No labeling of Ift20 was observed at the cilia or ciliary basal body in both the control and PD-L1 knockdown cells, which may likely be a paraformaldehyde fixation artifact as previously reported [27]. Ift20 is associated with Rab8, which is also associated with the BBSome through its guanine nucleotide exchange factor, Rabin8 [41]. We found that depletion of PD-L1 increased the population of cilia positive for Rab8a (Figure 3C,D) and BBS5 (Figure 3E,F) in NIH3T3 cells. These results suggest that PD-L1 modulates the Golgi accumulation of Ift20 and the ciliary localization Rab8a and BBS5.

### 3.5. Depletion of PD-L1 Increases Ciliary PC-2

**Figure 4:**
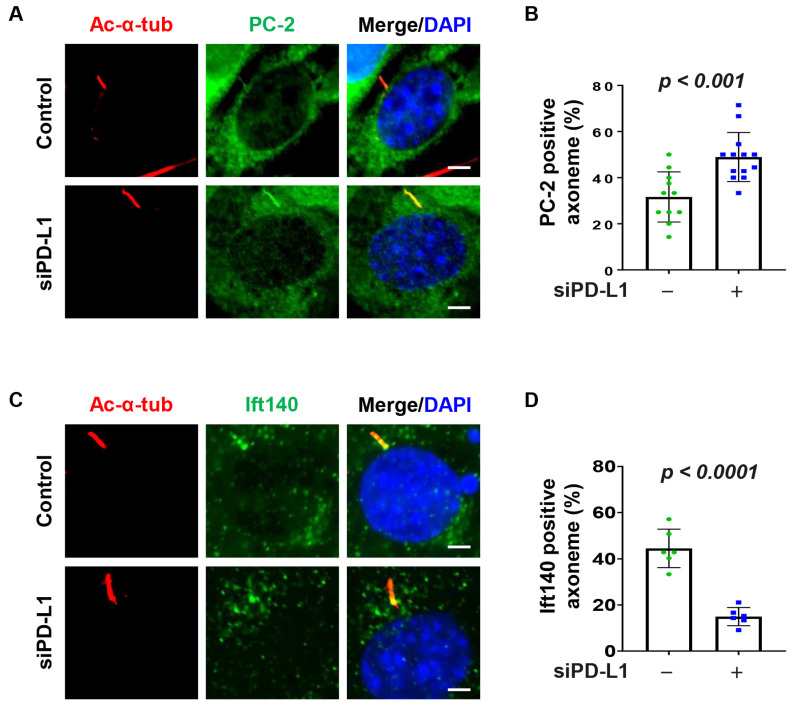
PD-L1 affects the ciliary recruitment of PC-2 and Ift140. (**A**) NIH3T3 cells co-stained for acetylated-α-tubulin and the PC-2 (polycystin 2) (green), in PD-L1 siRNA knockdown NIH3T3 cells compared to control siRNA cells. (**B**) Quantitative data of PC-2-positive cilia in PD-L1 siRNA knockdown NIH3T3 cells compared to control siRNA cells (*n* > 80). (**C**) NIH3T3 cells co-stained for acetylated-α-tubulin and the Ift140 (green), in PD-L1 siRNA knockdown NIH3T3 cells compared to control siRNA cells. (**D**) Quantitative data of Ift140-positive cilia in PD-L1 siRNA knockdown NIH3T3 cells compared to control siRNA cells (*n* > 80). All cells were counterstained with DAPI (blue). Scale bars, 20 μm.

Ift20, Rab8 and the BBSome regulate the import of ciliary sensory receptors [42]. For example, studies have found that BBS5 plays a role in polycystin 2 (PC-2) ciliary accumulation [43]. We therefore asked whether PD-L1 could modulate PC-2 ciliary accumulation. We found that depletion of PD-L1 increased the percentage of cilia positive for PC-2 in NIH3T3 cells (Figure 4A,B) and RCTE cells (Supplemental Figure S5A,B). We next investigate whether the accumulation of ciliary proteins observed in PD-L1 knockdown cells is associated with ciliary IFT-associated proteins known to regulate cilia trafficking dynamics. We found that knockdown of PD-L1 decreased the ciliary localization of Ift140 in NIH3T3 cells (Figure 4C,D), and in PD-L1 knockdown RCTE cells (Supplemental Figure S5C,D). Together, these results suggest that PD-L1-mediated PC-2 ciliary accumulation may be caused by a defective IFT-associated mechanism. 

### 3.6. PD-L1 Regulates PC-2 Ciliary Localization in a BBS5 Independent Manner

**Figure 5:**
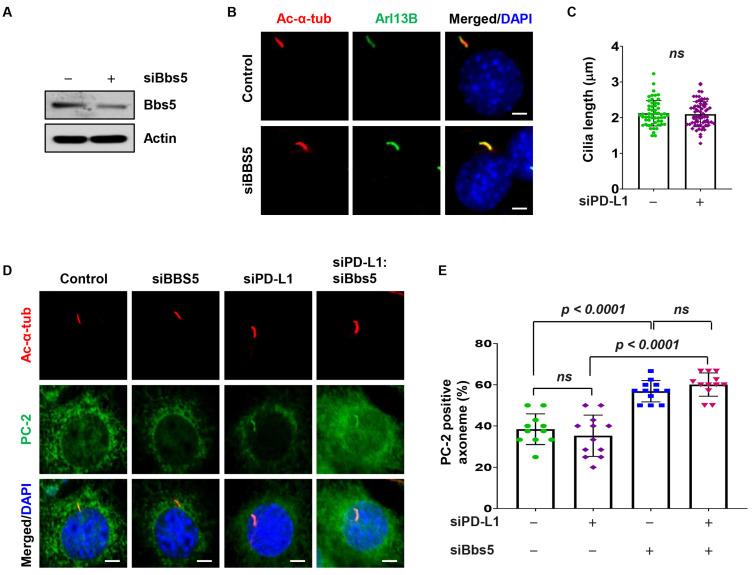
PD-L1 regulates PC-2 cilia localization in a BBS5-independent manner. (**A**) Western blot analysis evaluating the protein level of BBS5 after siRNA knockdown in NIH3T3 cells compared to control siRNA cells. (**B**) Representative images of acetylated-α-tubulin (red) co-stained with Arl13B (green) in BBS5 siRNA knockdown NIH3T3 cells compared to control siRNA cells. (**C**) Quantitative data of cilium length in BBS5 siRNA knockdown NIH3T3 cells compared to control siRNA cells (*n* > 100). (**D**) Representative images of acetylated-α-tubulin (red) co-stained with PC-2 (green) in PD-L1 and BBS5 single knockdown, and PD-L1:BBS5 siRNA double knockdown in 3T3 cells compared to control siRNA cells. (**E**) Quantitative data of PC-2-positive cilia in PD-L1 and BBS5 single knockdown, and PD-L1:BBS5 siRNA double knockdown in 3T3 cells compared to control siRNA cells (*n* > 75). All cells were counterstained with DAPI (blue). “ns” implies not significant. Scale bars, 20 μm.

We further investigated PD-L1’s role in PC-2 ciliary accumulation. Since BBSome proteins have been reported to regulate PC-2 ciliary accumulation [43], we hypothesized that PD-L1 may be modulating PC-2 ciliary accumulation via BBS5. We found that knockdown of BBS5 did not affect cilia length in NIH3T3 cells (Figure 5A–C). Next, we assessed the effect of double knockdown of PD-L1 and BBS5 on PC-2 ciliary localization. Compared to siRNA control, we found that knockdown of BBS5 alone did not affect the percentage of cilia positive for PC-2 in NIH3T3 cells (Figure 5D,E). In addition, double knockdown of PD-L1 and BBS5 did not affect the percentage of PC-2-positive cilia compared to knockdown of PD-L1 alone but increased the percentage of PC-2-positive cilia compared to knockdown of BBS5 alone (Figure 5D,E). These results suggest that PD-L1-mediated PC-2 ciliary accumulation is independent of BBS5.

### 3.7. PD-L1 Forms a Complex with BBS5 and PC-2

**Figure 6:**
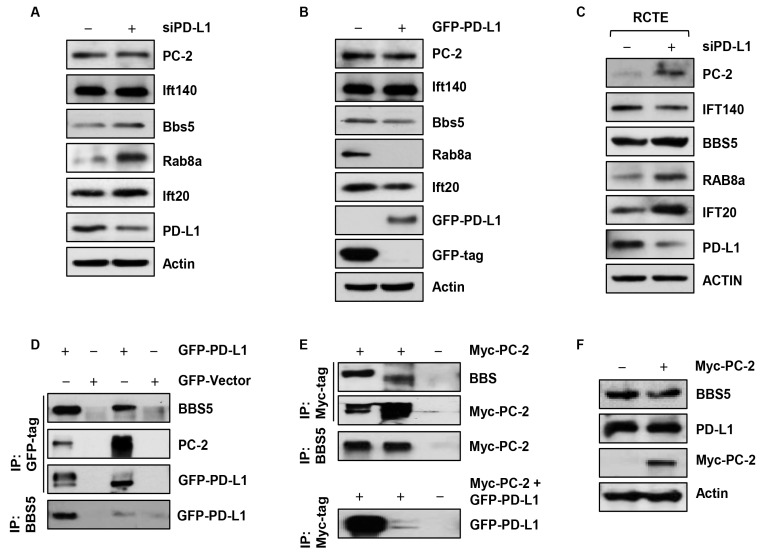
PD-L1 interacts with BBS5 and PC-2. (**A**) Western blot analysis of the expression of PC-2, Ift140, BBS5, Rab8a and Ift20 in PD-L1 siRNA knockdown NIH3T3 cells compared to control siRNA cells. (**B**) Western blot analysis of the expression of PC-2, Ift140, BBS5, Rab8a and Ift20 in GFP-PD-L1 overexpressed NIH3T3 cells compared to GFP-vector control cells. (**C**) Western blot analysis of the expression of PC-2, IFT140, BBS5, RAB8a and IFT20 in PD-L1 siRNA knockdown RCTE cells compared to control siRNA cells. (**D**) Western blot of the co-immunoprecipitation analysis between GFP-PD-L1 and BBS5, and PC-2, in HEK293T cells. (**E**) Western blot of the co-immunoprecipitation analysis between Myc-PC-2 and BBS5 and Myc-PC-2 and GFP-PD-L1 in HEK293T cells. (**F**) Western blot analysis of the expression of PD-L1 and BBS5 in Myc-PC-2 overexpressed HEK293T cells.

We next investigated whether the alterations in cilia localization of Rab8a, BBS5, PC-2 and Ift140, and the Golgi accumulation of Ift20, were associated with the expression levels of these proteins. We found that depletion of PD-L1 resulted in the upregulation of Rab8a, BBS5, and Ift20 in NIH3T3 cells (Figure 6A) and overexpression of GFP-PD-L1 resulted in their downregulation (Figure 6B). However, no significant changes were observed in the expression levels of PC-2 and Ift140 in NIH3T3 cells (Figure 6A,B). We further found that knockdown of PD-L1 resulted in the upregulation of Rab8a, BBS5, PC-2, and Ift20, and in the downregulation of Ift140 in RCTE cells (Figure 6C). Next, we evaluated the relationship between PD-L1 and the individual cilia proteins, including Rab8a, BBS5, PC-2, Ift20, and Ift140, by performing a series of immunoprecipitation experiments. We found that GFP-PD-L1 could pull down BBS5 and PC-2 (Figure 6D). In addition, we found that Myc-tagged PC-2 (Myc-PC-2) could also pull down BBS5 and GFP-PD-L1 (Figure 6E). Likewise, BBS5 could pull down GFP-PD-L1 and Myc-PC-2 (Figure 6D, E). Overexpression of Myc-PC-2 however, had no effect on their protein levels of PD-L1 and BBS5 (Figure 6F). Together, these results suggest that PD-L1 contributes to the regulation of Rab8a, BBS5, PC-2, Ift20, and Ift140 protein levels in a cell-type-dependent manner and that PD-L1 may physically form a complex with BBS5 and PC-2.

### 3.8. PD-L1 Regulates the Activation of Hh Signaling Pathway

**Figure 7:**
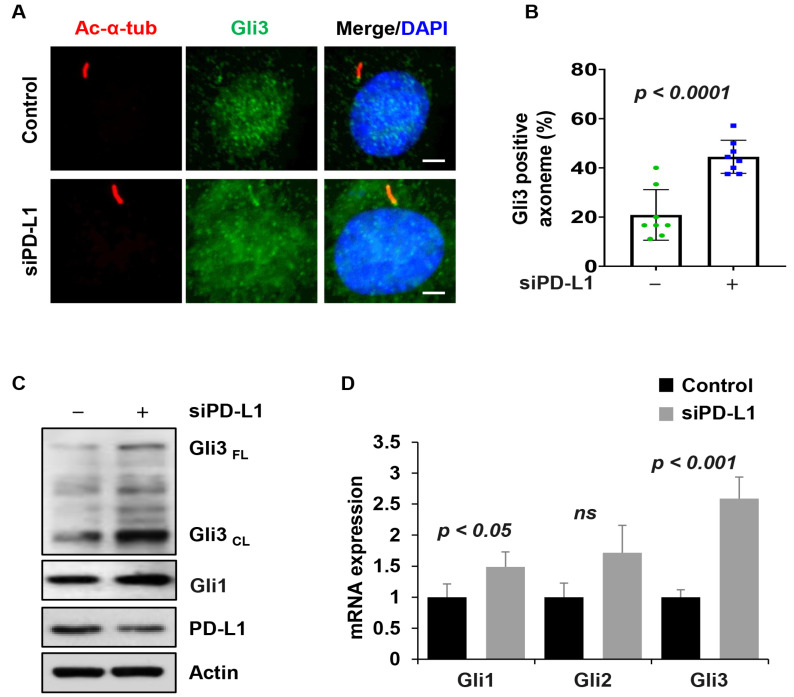
PD-L1 regulates Hedgehog signal transduction. (**A**) Representative images of acetylated-α-tubulin (red) co-stained with Gli3 (green), in PD-L1 siRNA knockdown NIH3T3 cells without SAG stimulation, compared to control siRNA cells (*n* > 100). All cells were counterstained with DAPI (blue). Scale bars, 20 μm. (**B**) Quantitative data of Gli3-positive cilia in unstimulated PD-L1 siRNA knockdown NIH3T3 cells compared to control siRNA cells. (**C**) Western blot analysis of the protein levels of Gli3 and Gli1 in unstimulated PD-L1 siRNA knockdown NIH3T3 cells compared to control siRNA cells. (**D**) qRT-PCR analysis of Hh signaling mediators (Gli1, Gli2 and Gli3) in unstimulated PD-L1 siRNA knockdown NIH3T3 cells compared to control siRNA cells. “ns” implies not significant.

Next, we analyzed whether PD-L1 influences cilia function, by focusing on the Hh signaling pathway reported to depend on primary cilia, regardless of the cilia length, for its activation [13]. Hh signaling is associated with increased levels of pathway mediators along the primary cilium including Gli2 and Gli3 [44,45]. We therefore evaluated whether the cilia localization of Hh signaling components was affected in the cilia of PD-L1 knockdown NIH3T3 cells. Under base conditions, the results indicated that the percentage of cilia positive for Gli3 (Figure 7A,B), and the protein level of Gli3 (Figure 7C) increased in PD-L1 knockdown cells compared to siRNA control cells. Surprisingly, we found that Gli1 expression was slightly upregulated at the protein and mRNA levels in PD-L1 knockdown cells (Figure 7C,D). Upon stimulation with SAG, the percentage of cilia positive for Gli3 (Supplemental Figure S6A,B), and the protein level (Supplemental Figure S6C) increased in PD-L1 knockdown cells, compared to siRNA control cells, but there was no change in its mRNA levels (Supplemental Figure S6D). Furthermore, Gli1 was downregulated at the protein and mRNA levels (Supplemental Figure S6C,D). These results suggest that at the basal state, PD-L1 derepresses the outcome of Hh signaling, whereas, upon SAG activation, the repression of the Hh signaling outcome is less.

## 4. Discussion

**Figure 8:**
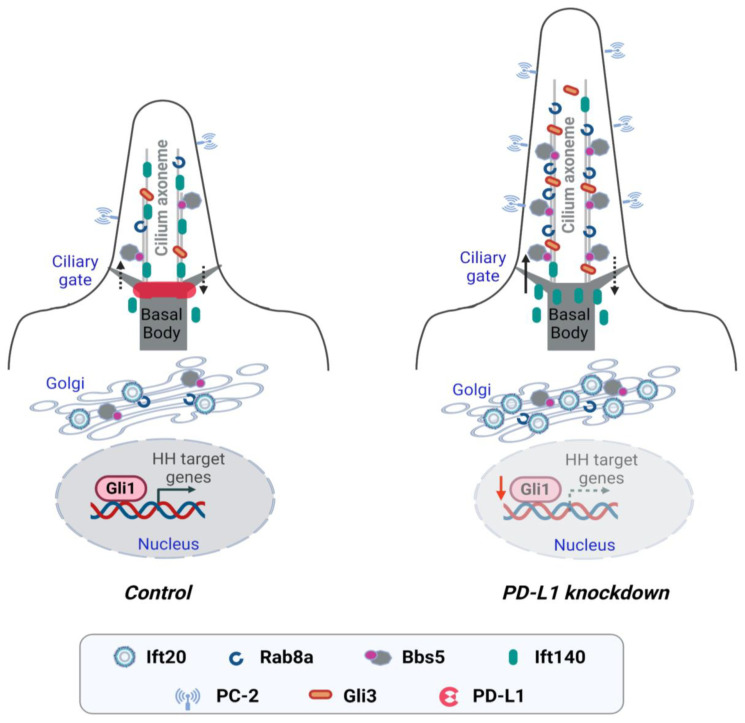
Working model for PD-L1 in the regulation of ciliogenesis. Enriched expression of receptors on the ciliary membrane makes the primary cilium a specialized organelle for receiving and transducing extracellular stimuli into cells. Vesicles carrying ciliary proteins leave the Golgi and move toward the basal body of the primary cilium. Active forms of Rab8, a master modulator for the ciliary protein trafficking, and the BBSome complex, regulates the entry of protein cargo to the cilium. The activities and basal body localization of Rab8 are modulated by Golgi-associated Ift20 and the BBSome. Knockdown of PD-L1 enhances ciliogenesis by modulating the localization of proteins important for ciliary protein sorting, trafficking, and cilia sensory signaling. Depletion of PD-L1 increases cilia length and increases the ciliary recruitment of Rab8a and BBS5. Also, knockdown of PD-L1 increases the ciliary localization of cilia sensory receptor PC-2, and Gli3 which results in the repression of the Hh signaling pathway.

The primary cilium, previously thought to be a rudimentary organelle has become an important signaling hub of renewed focus since defects in its structure or function are associated with a group of human disorders, collectively known as ciliopathies [6]. In recent years, studies have shown that ciliary proteins relocate to extraciliary sites and carry out functions other than ciliogenesis, including the formation of the immunological synapse [32]. However, whether regulators of the immune synapse participate in ciliogenesis is unknown. In this study, we investigated for the first time the role of the immune checkpoint protein and immunological synapse regulator PD-L1 on ciliogenesis. We found that PD-L1 was localized to the centrosome and the Golgi complex. Knockdown of PD-L1 increased cilia length and enhanced the ciliary accumulation and expression of proteins important for cilia assembly and trafficking (Ift20, Rab8a and BBS5), and cilia sensory function (PC-2 and Gli3) (Figure 8). Furthermore, we show an association between PD-L1 and BBS5, and PD-L1 and PC-2. We propose a model whereby PD-L1 modulates the localization and sorting of specific cargoes that contribute to the regulation of cilia biogenesis and function. 

We have shown the centrosomal association of endogenous PD-L1 by co-localization under both normal growth conditions and serum starvation. The centrosomal association of PD-L1 is also supported by immunofluorescent staining experiments with PD-L1 enrichment at the spindle poles during mitosis, a characteristic of known centrosomal proteins [46,47]. Overexpressed GFP-PD-L1, however, was not found at the centrosome. One possibility to explain this discrepancy is that the centrosome binding of the endogenous PD-L1 protein may be controlled by a partner protein that is present at low concentrations. The speculated partner may probably be a motor protein that concentrates PD-L1 at the site, rather than a permanent component of the centrosome because the endogenous localization of PD-L1 is affected by the disruption of microtubules. We also observed some staining reminiscent of the Golgi, raising the possibility that PD-L1 may be associated with a membranous structure close to the centrosomes. 

In a previous study, knockout of PD-L1 was reported to cause chromosomal defects [48]; however, the mechanisms were not fully understood. In this study, we identified PD-L1 at the centrosome and the spindle poles during mitosis, and its depletion resulted in centrosome amplification and cytokinesis defects. During cell division, the centrosome functions as the major microtubule-organizing center to ensure proper chromosome segregation. The presence of PD-L1 at the centrosome and spindle pole may, therefore, contribute to maintaining the functional properties and mechanisms associated with centrosome integrity, which when lost leads to centrosome amplification and culminates in chromosomal instability. 

Ciliary vesicle formation and protein trafficking are important steps in the hierarchy of cilia assembly. Proteins connected to ciliary vesicle formation, such as Golgi-associated Ift20, recruit Rab8 to ciliary vesicles [18] and promote the efficiency with which proteins such as PC-2 [49], are transported through the Golgi complex [50]. Since PD-L1 modulates the expression and localization of Ift20, Rab8a, and PC-2, we therefore speculate that one possible function of PD-L1 is to associate with and curb the transport of vesicles that contain proteins destined for the ciliary membrane. Depletion of PD-L1, liberates these vesicles, enhancing their delivery to the cilium.

The execution of cilia sensory capacities requires that signaling cascades are generated from sensory receptors to elicit appropriate responses. The presence of the polycystins in cilia of renal epithelial cells, for example, regulates calcium influx and their dysfunction leads to aberrant calcium influx and uncontrolled cyst formation [51], and retention of Hh receptors in embryo cilia could lead to various developmental manifestations [52]. In this study, we found that knockdown of PD-L1 resulted in the increased recruitment of sensory receptor PC-2 to the cilium. Moreover, PD-L1 formed a complex with PC-2. In this regard, PD-L1 may be a central player in a negative feedback mechanism to restrain PC-2-dependent ciliary signaling, ensuring adequate sensory outputs in ciliated cells.

Disruption of the proper functioning of the IFT machinery may lead to the inability to transduce or cause inappropriate activation of the Hh pathway [53,54]. In this work, we show that PD-L1-depleted cells have defects in the Hh signal transduction cascade. We observed that PD-L1-depleted cells have increased levels of the full-length Gli3 transcription factor in the absence of pathway stimulation. Unexpectedly, PD-L1-depleted cells showed slightly higher levels of Gli1 expression. This suggests that the increased level of full-length Gli3 in PD-L1-depleted cells may possess the ability to activate Gli1 expression under basal conditions, as opposed to the inhibition of Gli1 observed during pathway stimulation with Smoothened agonist, SAG. These observations suggest that there may be a role for PD-L1 in the non-canonical, and canonical signal transduction of the Hh pathway. 

Based on the literature, PD-L1 can be found in various subcellular localizations such as cytoplasmic, nuclear, and membrane-bound regions [55], and is most known for the role it plays in immune response and its use in immunotherapy [56]. Our characterization of PD-L1 in mammalian ciliated fibroblast and epithelial cells has revealed a potentially novel centrosomal localization of PD-L1; however, it is important to validate this localization by either using CRISPR knockout technology or primary cells from PD-L1 knockout mice. Our characterization of PD-L1 in mammalian ciliated fibroblast and epithelial cells has also revealed a novel function for PD-L1 and prompts new questions worth exploring. We found that ciliation, associated with recruitment of ciliary proteins, was enhanced in PD-L1 knockdown cells. However, it is quite possible that detailed studies, for example, using electron microscopy, may reveal other specific steps in ciliogenesis affected by PD-L1. Such studies are technically challenging and time-consuming due to the small size of cilia and the incomplete ciliation of most cell types. Our results give us a better understanding of the roles of PD-L1 in primary cilia function that may be disrupted in the context of disease. Furthermore, this study may provide the foundation for understanding how PD-L1 immune therapy may affect the outcome of ciliopathy-related diseases. We focused on the use of NIH3T3 fibroblast and renal epithelial RCTE cells, in large part to allow comparisons and extrapolations of the role of PD-L1 in ciliopathies, such as PKD, but recognize the possibility of differences between our observations in normal cells and diseased or mutant cells, and in in vivo models.

In summary, we have demonstrated that PD-L1 contributes to both primary cilia biogenesis and function. The present study provides evidence that PD-L1 participates in different steps of protein transport for the assembly and function of primary cilia. Its localization to the Golgi and potentially to the basal body suggests that PD-L1 is in a unique position to direct movement of proteins destined for the ciliary membrane and into the cilium. 

## Data Availability

The authors declare that the data supporting the findings of this study are available within the article and the Supplementary Material, or from the corresponding author upon reasonable request.

## Supplementary Materials

The following supporting information can be downloaded at: https://www.mdpi.com/article/10.3390/cells13121003/s1, Figure S1: PD-L1 co-localizes with centrosome appendage proteins; Figure S2: Disruption of the Golgi disperses PD-L1 stacked structure; Figure S3: PD-L1 regulates genomic stability; Figure S4: PD-L1 regulates ciliogenesis in human epithelial cells; Figure S5: PD-L1 affects the ciliary recruitment of PC-2 and Ift140 in RCTE cells; Figure S6: PD-L1 represses Hedgehog signal transduction.
