# Supplementary material for: The Immune Checkpoint Protein PD-L1 Regulates Ciliogenesis and Hedgehog Signaling
Source: Cells. 2024 Jun 8;13(12):1003. doi: 10.3390/cells13121003 (PMC11201989; doi:10.3390/cells13121003)
Supplement: Supplementary file 1 [file cells-13-01003-s001.zip › cells-2946937-supplementary.pdf]

Supplemental Data

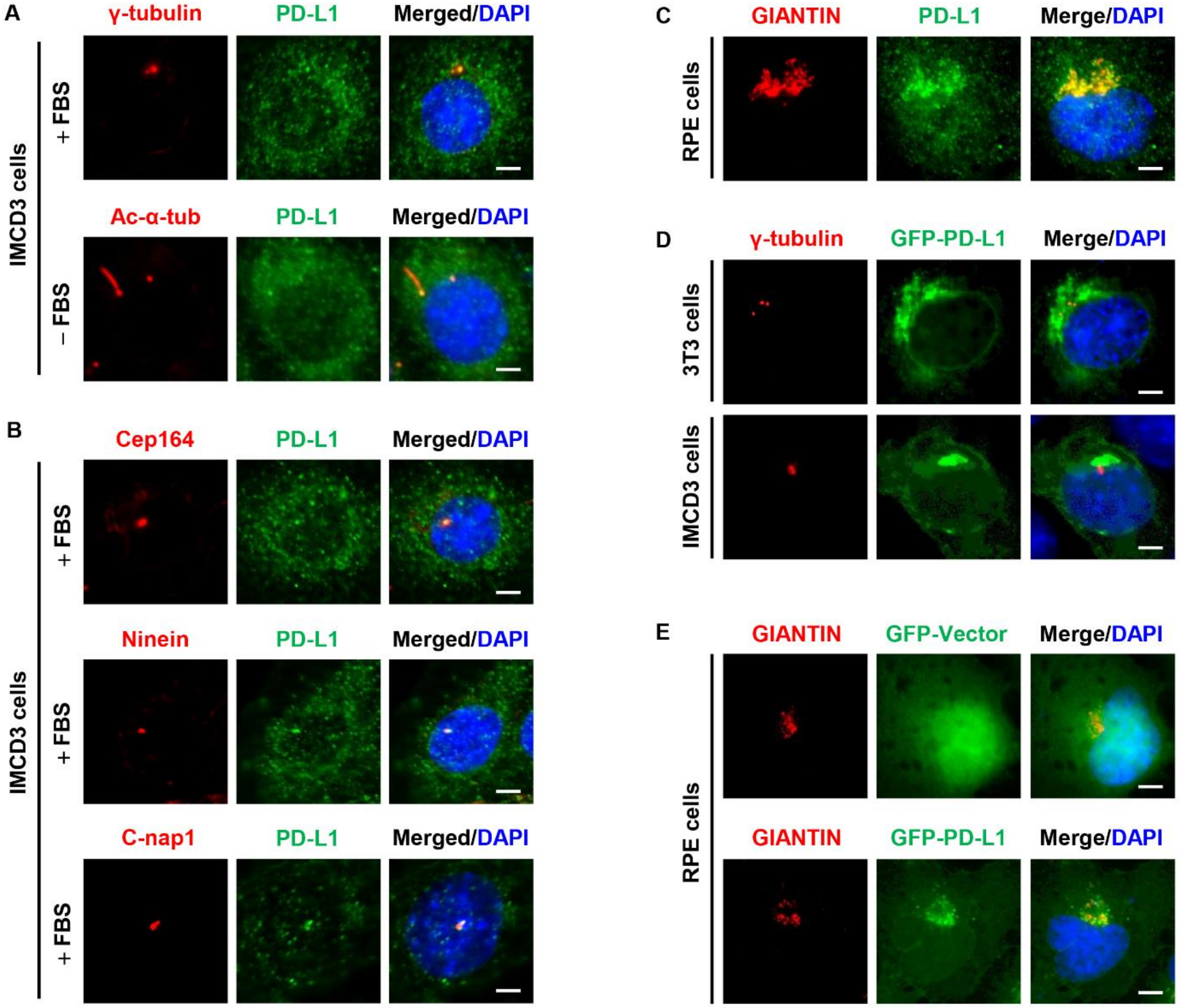

Supplemental Figure S1. PD-L1 co-localizes with centrosome appendage proteins. (A) mIMCD3 cells stained with PD-L1 (green) antibody and co-stained with centrosome marker, γ-tubulin (red) (top panel), and cilium marker, acetylated-α-tubulin (red) (bottom panel). (B) mIMCD3 cells co-stained with PD-L1 (green) and centrosome distal appendage marker Cep164 (red) (top panel), centrosome subdistal appendage marker Ninein (red) (middle panel), and centriole linker protein C-nap1 (red) (bottom panel). (C) RPE cells stained with PD-L1 (green) antibody and co-stained with Golgi marker, Giantin (red). (D) Overexpression of GFP-PD-L1 in NIH3T3 cells co-stained with γ-tubulin (red) (top panel), and mIMCD3 cells co-stained with γ-tubulin (red) (bottom panel).(E) Overexpression of GFP-PD-L1 co-stained with Golgi marker, Giantin in RPE cells and counterstained with DAPI (blue). All cells are counterstained with DAPI (blue). Scale bars, 20 μm.

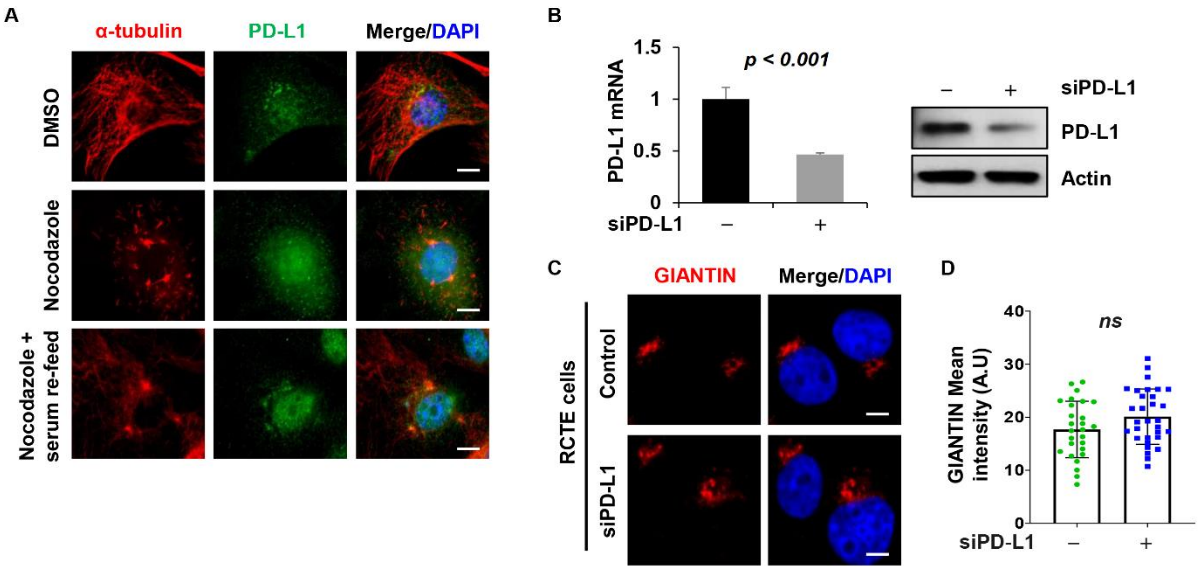

Supplemental Figure S2. Disruption of the Golgi disperses PD-L1 stacked structure. (A) NIH3T3 cells stained for PD-L1 (green), co-stained with α-tubulin (red) and counterstained with DAPI (blue) in nocodazole treated cells compared to control cells upon nocodazole release. (B) qRT-PCR and western blot analysis evaluating knockdown efficiency after PD-L1 siRNA knockdown in RCTE cells. (C) Representative images of Giantin (red), counterstained with DAPI (blue) in PD-L1 siRNA knockdown RCTE cells compared to control siRNA cells. (D) Quantitative data of fluorescence intensity of Giantin in PD-L1 siRNA knockdown RCTE cells compared to control siRNA cells. ($n>50$). "*ns*" implies not significant. Scale bars, 20 μm.

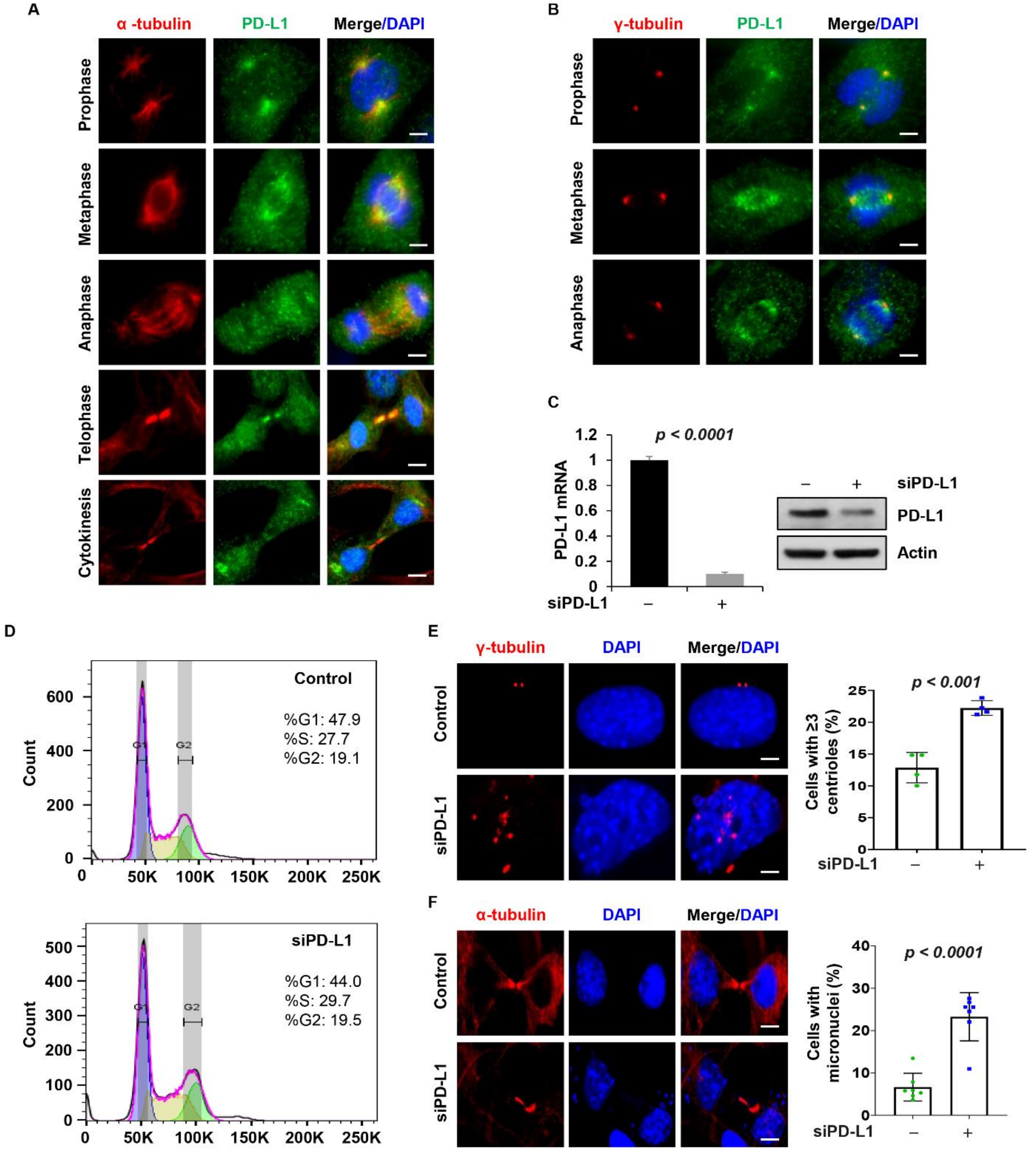

Supplemental Figure S3. PD-L1 regulates genomic stability. (A) Representative image of PD-L1 (green), co-stained with α-tubulin (red) during cell division in NIH3T3 cells. (B) Representative image of PD-L1 (green), co-stained with γ-tubulin (red), and counterstained with DAPI (blue) during cell division. (C) qRT-PCR and western blot analysis evaluating knockdown efficiency after PD-L1 siRNA knockdown in NIH3T3 cells. (D) Pictorial graph showing the proportion of cells in distinct phases of cell cycle in PD-L1 siRNA knockdown NIH3T3 cells compared to control siRNA cells. (E) Representative images of γ -tubulin (red) counterstained with DAPI (blue), and quantitative data of the percentage of cells with ≥3 centrosomes (*n*>*75*) in PD-L1 siRNA

knockdown NIH3T3 cells compared to control siRNA cells. (F) Representative images of α-tubulin (red) counterstained with DAPI (blue), and quantitative data of the percentage of cells with cytokinesis defects including micronuclei (both at interphase and at anaphase) (*n*>*150*) in PD-L1 siRNA knockdown NIH3T3 cells compared to control siRNA cells. Scale bars, 20 μm

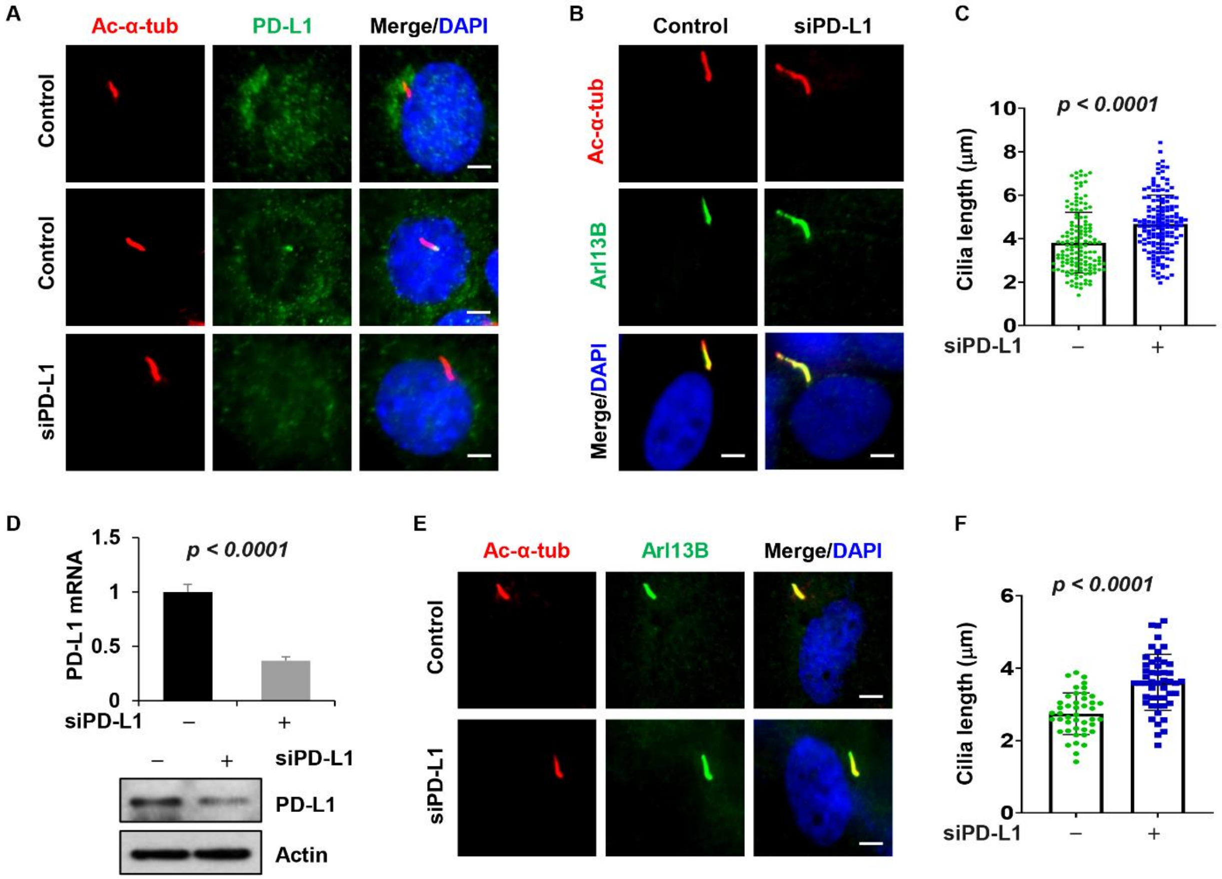

Supplemental Figure S4. PD-L1 regulates ciliogenesis in human epithelial cells. (A) Representative images of acetylated-α-tubulin (red) co-stained with PD-L1 (green), and counterstained with DAPI (blue), in PD-L1 siRNA knockdown NIH3T3 cells compared to control siRNA cells. (B) Representative images of acetylated-α-tubulin (red) co-stained with Arl13B (green), and counterstained with DAPI (blue) in PD-L1 siRNA knockdown RCTE cells compared to control siRNA cells. (C) Quantitative data of cilium length in PD-L1 siRNA knockdown RCTE cells compared to control siRNA cells (*n>100*). (D) qRT-PCR and western blot analysis evaluating knockdown efficiency after PD-L1 siRNA knockdown in RPE cells. (E) Representative images of acetylated-α-tubulin (red) co-stained with Arl13B (green), and counterstained with DAPI (blue) in PD-L1 siRNA knockdown RPE cells compared to control siRNA cells. (F) Quantitative data of cilium length in PD-L1 siRNA knockdown RPE cells compared to control siRNA cells (*n>100*). Scale bar, 20 µm.

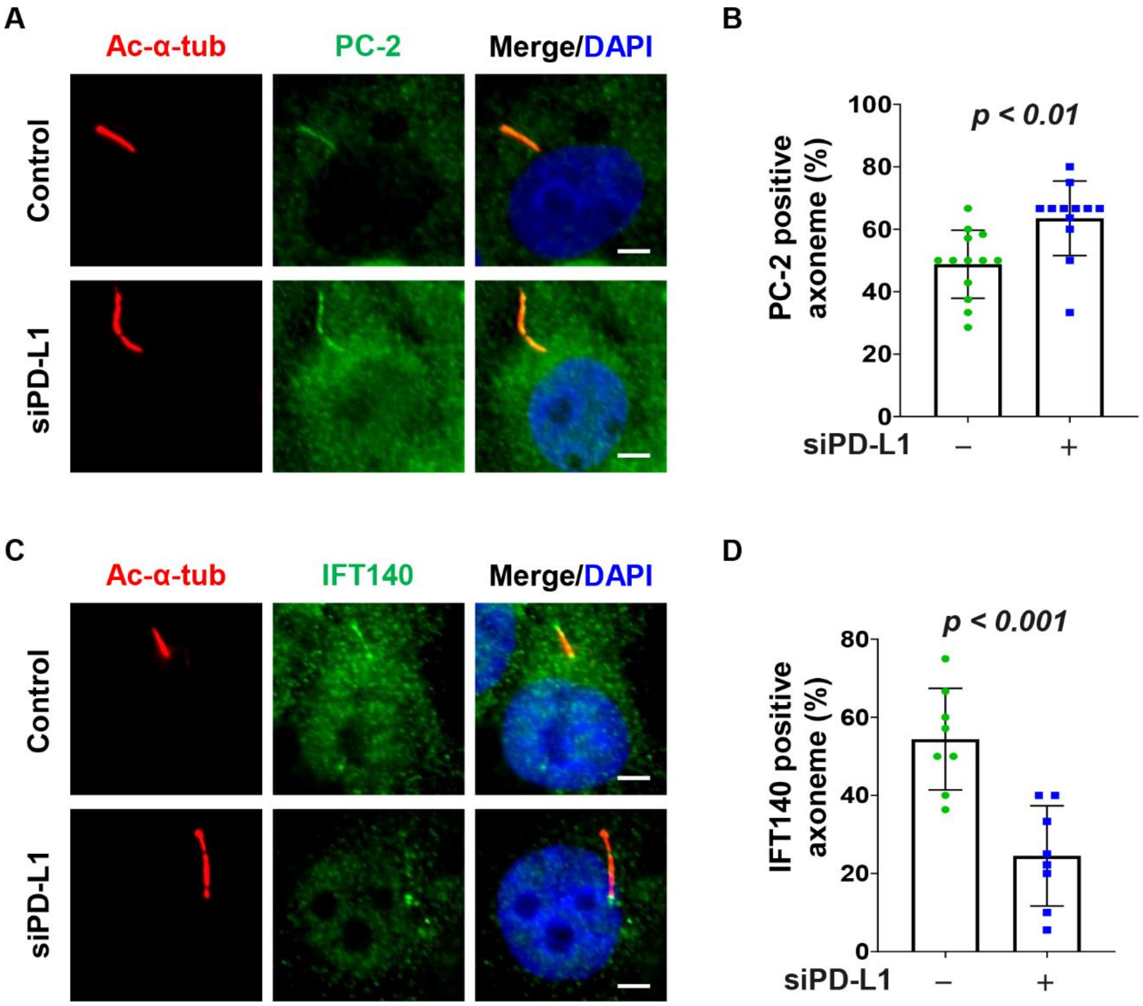

Supplemental Figure S5. PD-L1 affects the ciliary recruitment of PC-2 and Ift140 in RCTE cells. (A) Representative images of acetylated-α-tubulin (red), co-stained with PC-2 (polycystin 2)(green), and counterstained with DAPI (blue) in PD-L1 siRNA knockdown RCTE cells compared to control siRNA cells. (B) Quantitative data of PC-2 positive cilia in PD-L1 siRNA knockdown RCTE cells compared to control siRNA cells (*n>100*). (C) Representative images of acetylated-α-tubulin (red), co-stained with Ift140 (green), in PD-L1 siRNA knockdown RCTE cells compared to control siRNA cells. (D) Quantitative data of Ift140 positive cilia in PD-L1 siRNA knockdown RCTE cells compared to control siRNA cells (*n>100*). Scale bars, 20 μm.

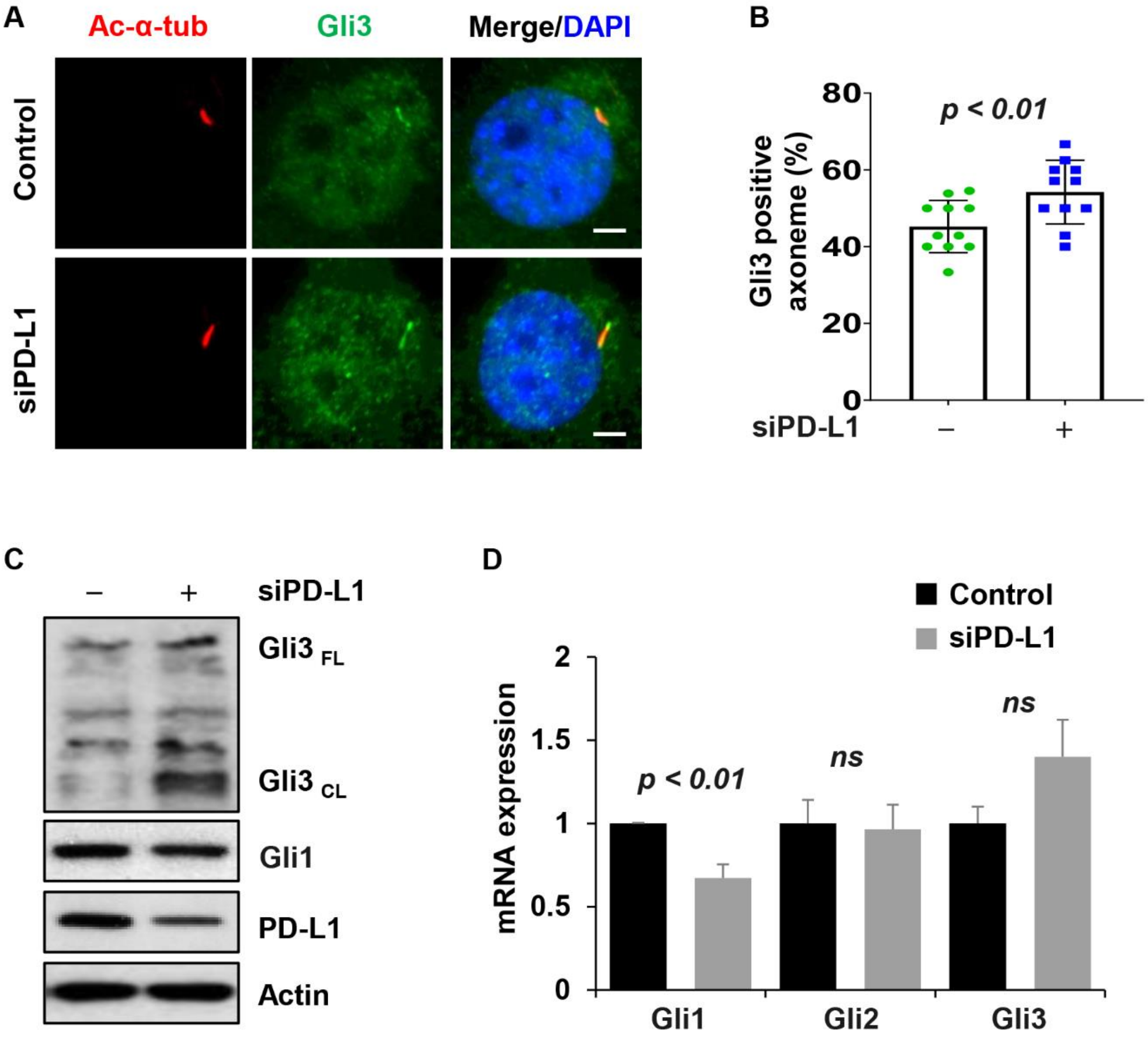

Supplemental Figure S6. PD-L1 represses Hedgehog signal transduction. (A) Representative images of acetylated-α-tubulin (red) co-stained with Gli3 (green), in PD-L1 siRNA knockdown SAG stimulated NIH3T3 cells, compared to control siRNA cells. All cells were counterstained with DAPI (blue). Scale bars, 20 μm. (B) Quantitative data of Gli3 positive cilia in SAG stimulated PD-L1 siRNA knockdown NIH3T3 cells compared to control siRNA cells (*n>100*). (C) Western blot analysis of the protein levels of Gli3 and Gli1 in SAG stimulated PD-L1 siRNA knockdown NIH3T3 cells compared to control siRNA cells. (D) qRT-PCR analysis of Hh signaling mediators (Gli1, Gli2 and Gli3) in SAG stimulated PD-L1 siRNA knockdown NIH3T3 cells compared to control siRNA cells. "*ns*" implies not significant.
